# Exploring maladaptive early schemas in adults with bipolar disorder

**DOI:** 10.1192/j.eurpsy.2024.899

**Published:** 2024-08-27

**Authors:** Z. Bencharfa, H. Ballouk, I. Katir, F. Laboudi, A. Ouanass

**Affiliations:** Ar-razi Psychiatric Hospital, Salé, Morocco

## Abstract

**Introduction:**

Bipolar disorder is a severe and chronic mental pathology, with an estimated prevalence of 1% in the general population. It is a complex pathology, encompassing a wide variety of severe and contradictory symptoms, with harmful repercussions on the patient’s personal, emotional, social, professional and conjugal life, precipitating relapse. By improving our knowledge of bipolar disorder, we can support and accompany patients, helping them to understand their illness, to be able to manage it, to resolve the problems that may arise from it, and to prevent relapses and the occurrence of further episodes.

**Objectives:**

The aim of our work is to explore maladaptive early patterns in people with bipolar disorder in the intercritical period in relation to their symptomatology and functional disability, given that consideration of maladaptive early patterns (IAPs) could lead to better identification, understanding and management of bipolar disorder.

**Methods:**

We conducted a cross-sectional, descriptive and analytical study. The sample in our study consisted of 40 bipolar adults and 40 control adults, recruited from the various inpatient and outpatient departments of our hospital. They were all university graduates, aged between 20 and 60, followed for at least 06 months and stabilized on treatment. After collecting the various socio-demographic and clinical data, we used the Young schema questionnaire-short form (YSQ-S1).

**Results:**

Our study sample seemed to be characterized by certain specificities: high “self-sacrifice”, “high demands” and “exaggerated personal rights”. Feelings of dependence and incompetence were also high among our patients, especially those with type I bipolar disorder, leading to a marked decline in self-esteem and autonomy.

**Conclusions:**

The data we have retained from this work show us the importance of drug, psychotherapeutic and family management in achieving thymic stability and psychological and relational well-being.

**Disclosure of Interest:**

None Declared

